# Reprogramming pancreatic stellate cells via p53 activation: A putative target for pancreatic cancer therapy

**DOI:** 10.1371/journal.pone.0189051

**Published:** 2017-12-06

**Authors:** Maya Saison-Ridinger, Kathleen E. DelGiorno, Tejia Zhang, Annabelle Kraus, Randall French, Dawn Jaquish, Crystal Tsui, Galina Erikson, Benjamin T. Spike, Maxim N. Shokhirev, Christopher Liddle, Ruth T. Yu, Michael Downes, Ronald M. Evans, Alan Saghatelian, Andrew M. Lowy, Geoffrey M. Wahl

**Affiliations:** 1 Gene Expression Laboratory, Salk Institute for Biological Studies, La Jolla, California, United States of America; 2 Clayton Foundation Peptide Biology Lab, Helmsley Center for Genomic Medicine, Salk Institute for Biological Studies, La Jolla, California, United States of America; 3 Department of Surgery, Division of Surgical Oncology, Moores Cancer Center, University of California, San Diego, La Jolla, California, United States of America; 4 Integrative Genomics and Bioinformatics Core, Salk Institute for Biological Studies, La Jolla, California, United States of America; 5 Huntsman Cancer Institute, Department of Oncologic Sciences, University of Utah, Salt Lake City Utah, United States of America; 6 Storr Liver Centre, Westmead Institute for Medical Research and Sydney Medical School, University of Sydney, Westmead Hospital, Westmead, New South Wales, Australia; Mayo Clinic Rochester, UNITED STATES

## Abstract

Pancreatic ductal adenocarcinoma (PDAC) is characterized by an extremely dense fibrotic stroma, which contributes to tumor growth, metastasis, and drug resistance. During tumorigenesis, quiescent pancreatic stellate cells (PSCs) are activated and become major contributors to fibrosis, by increasing growth factor signaling and extracellular matrix deposition. The p53 tumor suppressor is known to restrict tumor initiation and progression through cell autonomous mechanisms including apoptosis, cell cycle arrest, and senescence. There is growing evidence that stromal p53 also exerts anti-tumor activity by paracrine mechanisms, though a role for stromal p53 in PDAC has not yet been described. Here, we demonstrate that activation of stromal p53 exerts anti-tumor effects in PDAC. We show that primary cancer-associated PSCs (caPSCs) isolated from human PDAC express wild-type p53, which can be activated by the Mdm2 antagonist Nutlin-3a. Our work reveals that p53 acts as a major regulator of PSC activation and as a modulator of PDAC fibrosis. In vitro, p53 activation by Nutlin-3a induces profound transcriptional changes, which reprogram activated PSCs to quiescence. Using immunofluorescence and lipidomics, we have also found that p53 activation induces lipid droplet accumulation in both normal and tumor-associated fibroblasts, revealing a previously undescribed role for p53 in lipid storage. *In vivo*, treatment of tumor-bearing mice with the clinical form of Nutlin-3a induces stromal p53 activation, reverses caPSCs activation, and decreases fibrosis. All together our work uncovers new functions for stromal p53 in PDAC.

## Introduction

Pancreatic ductal adenocarcinoma (PDAC) represents the third leading cause of cancer-related deaths in the United States, with an overall 5-year survival rate of ~9% [1]. Late diagnosis and limited response to therapy are major contributing factors to this dismal prognosis. PDAC is characterized by the formation of an extremely dense fibrotic stroma (also called desmoplasia) that surrounds the cancer cells and represents up to 90% of the tumor volume [2]. This reactive stroma has been implicated as a major facilitator of tumor growth, metastasis and drug resistance [3]. The stroma is composed of extracellular matrix (ECM) proteins and a diverse population of cells including inflammatory cells and fibroblasts [4]. Pancreatic stellate cells (PSCs) are the resident fibroblasts of the pancreas and represent the principal source of fibrosis in PDAC when activated [5]. In healthy tissues, PSCs are quiescent, characterized by the presence of cytoplasmic lipid droplets rich in vitamin A, and play a role in normal tissue architecture by regulating ECM turnover [6]. During tumor progression, PSCs are activated by pro-inflammatory cytokines, growth factors, and other oxidative and/or metabolic stresses, and transdifferentiate into myofibroblast-like cells [7]. This activation is accompanied by a loss of cytoplasmic lipid droplets, increased expression of the cytoskeletal protein α-smooth muscle actin (αSMA), and an increase in proliferation [8]. Cancer-associated PSCs (caPSCs) establish fibrosis via the synthesis of excessive ECM proteins (i.e. collagen, fibronectin, laminin) and ECM remodeling proteins (matrix metalloproteinases and their inhibitors). This dense ECM compresses intra-tumoural vasculature, causing hypoxia and impeding drug delivery to the tumor [9,10]. Moreover, caPSCs secrete growth factors and cytokines that sustain their own activation and promote cancer cell growth, survival and migration [11,12].

Based on the important role of the stroma in PDAC, therapeutic strategies targeting caPSCs or enzymatically digesting the ECM have emerged and have shown encouraging results in preclinical models [13,14]. Sherman et al., demonstrated that reverting caPSC activation using a vitamin D analogue resulted in reduced tumor growth, improved response to chemotherapy and increased survival [14]. However, recent studies report that depletion of stromal cells favors tumor aggressiveness and decreases survival in mice [15,16]. These findings suggest that caPSC reprogramming, rather than depletion, might be a better stromal targeting strategy for improving therapeutic outcomes.

Here, we explore the possibility that p53 pathway activation may provide another mechanism for reprogramming the activated pancreatic cancer stroma. The p53 protein is a sequence-specific transcription factor that functions as a major tumor suppressor [17]. Upon oncogenic stress, p53 is activated to induce a variety of context-dependent programs including cell cycle exit, apoptosis, or replicative senescence, in order to restrict malignant cell propagation [18]. Consequently, p53 activity is often mitigated in tumor cells either by mutation of the gene itself, or by alteration of p53 regulators [19]. More recent studies have reported that stromal p53 also exerts antitumor activity by paracrine mechanisms [20]. Thus, p53 ablation in stromal cells can promote tumor cell proliferation [21], angiogenesis [22], invasion and metastasis [23,24]. Furthermore, genetic deletion of p53 in the stromal compartment promotes tumor initiation and progression *in vivo* [25,26]. These observations suggest that attenuation of p53 activity in the tumor stroma may favor tumor development. While most evidence suggests that the fibroblastic stromal cells encode wild type p53 [27], recent studies suggest that cancer cells acquire the ability to suppress stromal p53 function via paracrine mechanisms [28,29]. These observations suggest that activating the p53 pathway in caPSC may compromise the tumor-supportive functions of activated stroma.

p53 activation can be achieved through the use of non-genotoxic Mdm2 antagonists. Mdm2 is an E3 ubiquitin ligase that negatively regulates p53 by targeting p53 for ubiquitin-dependent degradation and by inhibiting its transactivation function [30]. Nutlins are Mdm2 antagonists that disrupt p53-Mdm2 binding, thereby stabilizing and activating p53 [31]. We used the Mdm2 antagonist Nutlin-3a and its clinically related derivative RG7112 to determine the effects of p53 activation in caPSCs *in vitro* and *in vivo*, respectively. Our work reveals that Nutlin-3a induces p53 activation in both murine and human caPSCs and reprograms these cells towards quiescence. This quiescent state is characterized by decreased proliferation, downregulation of αSMA expression, and accumulation of lipid droplets. Our study reveals that p53 activation directly induces lipid droplet accumulation and an increase in lipid droplet-associated lipids in caPSCs and fibroblasts from other tissues. Finally, we show that RG7112, a p53 agonist currently in clinical trials, reverses caPSC activation *in vivo* and reduces desmoplasia in an allograft model of PDAC.

## Materials and methods

### Cell culture and drug preparation

Human primary PSC were isolated from PDAC surgical specimens using the outgrowth method [32], in accordance with a protocol approved by the Institutional Review Board at Moores Cancer Center, UC San Diego. Written informed consent was obtained and patients’ information was clearly anonymised. Briefly, tumors were dissected into small pieces (0.3–0.5 mm) and embedded in growth factor reduced Matrigel (Corning) on a 60-mm culture dish. Matrigel was submerged with medium and incubated for 6 days. Explants with fibroblast outgrowth were harvested, suspensed in PBS and incubated at room temperature with 0.025% tryspin for 15 min. Cells were collected by centrifugation and seeded on 10-cm plates with medium. caPSC identity was confirmed by immunostaining for αSMA (+), Vimentin (+), GFAP (+), Desmin (+) and Keratins (-). Primary caPSC are cultured for 10–15 passages before senescence occurs.

Mouse pancreatic stellate cells (PSCs) were isolated from the pancreata of 8-week old, wild-type C57B6/J mice as previously described [14]. Briefly, pancreatic tissue was minced and digested with 0.02% Pronase (Roche), 0.05% Collagenase P (Roche), and 0.1% DNase (Roche) in Gey’s balanced salt solution (GBSS, Sigma) at 37°C for 20 min. Digested tissue was then filtered through a 100 μm nylon mesh. Washed cells were resuspended in 9.5ml GBSS containing 0.3% bovine serum albumin (BSA, Sigma) and 8 ml of 28.7% Nycodenz solution (Sigma). The cell suspension was layered beneath GBSS containing 0.3% BSA, and centrifuged at 1400 x g for 25min at 4°C. The cells of interest were harvested from the interface of the Nycodenz solution and the aqueous solution. Isolated PSCs were washed with GBSS and resuspended in PSC media.

The primary skin fibroblast lines HF and 67LR were obtained from the laboratories of Jan Karlseder and Martin Hetzer (The Salk Institute, CA), respectively. The mouse pancreatic cancer line, FC-1199, isolated from PDAC in *LSL-Kras*^*G12D/+*^*; Trp53*^*R172H/+*^*; Pdx1-Cre* C57B6/J mice, was obtained from the David Tuveson laboratory (Cold Spring Harbor Laboratory, NY). The Retinal Pigmented Epithelial cell line (RPE) and the osteosarcoma cell line U2OS were purchased from the American Type Culture Collection (ATCC).

Primary human and mouse PSCs were cultured in DMEM (Fisher Scientific) supplemented with 20% FBS (Peak Serum), 100 mM Sodium Pyruvate (Life Technologies), 2 mM L-Glutamine (Life Technologies), 1x non-essential amino acids (Life Technologies) and antibiotics (penicillin 100 U/ml and streptomycin 100 mg/ml, Life Technologies). The other cell lines were cultured in DMEM with 10% FBS and antibiotics. RG7112 and its inactive enantiomer were provided by Roche (Basel, Switzerland), PD-332991 was provided by Pfizer (USA). Nutlin-3a and Nutlin-3b were synthesized following the protocol outlined in [33], by Sundia Meditech Company (Shanghai, China). The structure and purity of Nutlin-3a and Nutlin-3b (≥98.0%) were confirmed by NMR, HPLC, and LCMS analyses, as well as by carbon, hydrogen and nitrogen elemental analyses. All drugs were prepared as 10 mM stock solutions in DMSO. Drugs were diluted in media to the following final concentrations: Nutlin-3a 10 μM, Nutlin-3b 10 μM, PD-332991 0.5 μM, RG7112 as indicated in S4 Fig.

### Cloning, virus preparation and infection

The pLV-S16-p53dd vector expressing p53dd [34] was generated by cloning human p53dd (p53 aa 1–11,305–393) in frame behind Tag-BFP (Evrogen, Cat#26291) and cloning of the resulting fusion ORF downstream of the CMV promoter in the p156RRLsin backbone [35]. The 3rd generation pLV-S16-p53dd lentiviral vector was packaged by co-transfection with Lipofectamine 2000 (ThermoFisher) into HEK293T cells with the packaging plasmids pMD2.G, pRRE and pRSV/REV. Fresh medium was added 6 h after transfection and collected 48h later to be ultra-centrifuged at 24,000 rpm 4C for 2h. The pellet was re-suspended in DMEM-10% FBS and used to infect caPSC-82. Successful infection was monitored by visualization of the BFP using fluorescent microscopy (Leica). Over time, p53dd-expressing cells out-grew non-infected cells and all cells were identified as BFP-positive.

### Mice

Mice were housed in accordance with NIH guidelines in Association for Assessment and Accreditation of Laboratory Animal Care (AAALAC)-accredited facilities at the Salk Institute. All experimental protocols were approved by the Institutional Animal Care and Use Committee at the Salk Institute. The p53 knockout mice [36] were purchased from Jackson Laboratory and bred into a C57B6/J background. 8 to 12 week old wild-type male C57B6/J mice were orthotopically transplanted as described previously [37] with 10,000 FC-1199 cells. For in vivo experiments, the RG7112 compound was provided by Roche and prepared as recommended. On day 8, mice were treated by oral gavage daily with either vehicle (GTX004172) or RG7112 at 200 mg/kg. Mice were euthanized on day 24 and pancreatic tumors were harvested.

### RNA extraction and RT-qPCR

Cells were lysed in 1 mL of Trizol (Life Technologies) and total RNA isolation was performed using an RNeasy micro Kit (Qiagen, #74004) according to the manufacturer’s instructions. Cells sorted by flow-cytometry were pelleted and RNA isolation was performed using an Ambion RNAqueous®-Micro kit (ThermoFisher) per the manufacturer’s instructions. cDNA synthesis was carried out using iScript reagent (Bio-Rad), and real-time qPCR was performed using Power SYBR Green PCR Master Mix (Applied biosystems) on the ABI 7900 detection system (Applied Biosystems). Relative expression values were determined using the standard curve method. Results were normalized to the housekeeping gene Rplp0. Primer sequences can be found in S1 Table.

### Western-blot and antibodies

Tissues were dissected, flash frozen in liquid nitrogen, and homogenized in RIPA buffer containing protease inhibitors. Total protein from cell lysates was prepared as previously described [38]. Total protein was separated by SDS-PAGE then transferred to a PVDF membrane (Millipore, IPFL00010). Primary antibodies for human cells were anti-p53 (mouse monoclonal, OP43 Calbiochem, 1/1000), anti-p21 (rabbit polyclonal, sc-397 Santa-Cruz, 1/1000), anti-RB (mouse monoclonal, 9309 Cell Signaling, 1/1000), anti-pRB-Ser780 (rabbit monoclonal, 8180 Cell Signaling, 1/1000), anti-Actin (rabbit polyclonal, A2066 Sigma, 1/10000), and anti-Tubulin (rabbit polyclonal, 926–42211 Licor, 1/5000). Primary antibodies for mouse tissues were p53 (mouse monoclonal, 2524 Cell Signaling, 1/1000) and p21 (rabbit polyclonal, sc-397 Santa-Cruz, 1/1000). Secondary antibodies were conjugated to Alexa Fluor 680 (Life Technology) or IRDye 800 (LiCOR) for scanning in the LiCOR Odyssey system.

### Lipid extraction

Lipids were extracted using a modified version of the Bligh-Dyer method [39]. Briefly, cell pellets were manually shaken in a glass vial (VWR) with 1.5 mL citric acid buffer (100 mM trisodium citrate, 1 M NaCl [pH 3.6]), 1.5 mL methanol and 3.0 mL chloroform for 30s. The resulting mixture was vortexed for 15s and centrifuged at 2200 x g for 6 min to induce phase separation. The organic (bottom) layer was retrieved using a Pasteur pipette, dried under a gentle stream of nitrogen, and either reconstituted in chloroform immediately for LC/MS analysis or stored at -80°C and analyzed within one month.

### LC/MS

Lipidomic analysis was performed on an Ultimate 3000 uHPLC online with a Thermo q-Exactive Plus quadrupole-orbitrap mass spectrometer equipped with an electrospray ion source. Data were acquired in positive ionization mode. Solvent A consisted of 95:5 water:methanol, 5 mM ammonium formate with 0.1% formic acid. Solvent B was 60:35:5 isopropanol:methanol:water, 5 mM ammonium formate with 0.1% formic acid. A Luna (Phenomenex) C5 column (5 μm, 100 Å, 4.6 mm × 50 mm) equipped with a guard column (C4, 2 μm frit, 2.0 mm × 20 mm) was used. The gradient was held at 0% B between 0 and 5 min, raised to 20% B at 5.1 min, increased linearly from 20% to 100% B between 5.1 and 45 min, held at 100% B between 45 min and 53 min, returned to 0% B at 53.1 min, and held at 0% B until 60 min. Flow rate was 0.1 mL/min from 0 to 5 min, 0.4 mL/min between 5.1 min and 45 min, and 0.5 mL/min between 45.1 min and 60 min. Data were collected in full MS/dd-MS^2^ (top 8). Full MS was acquired for 150–2000 *m/z* with resolution of 70,000, target ion of 1x10^6^ and a maximum injection time of 100 ms. MS^2^ was acquired for 200–2000 *m/z* (fixed first mass *m/z* 50) with resolution of 35,000, a target ion of 1x10^5^ and a maximum injection time of 50 ms. Spray voltage was 4.0 kV. Sheath, auxiliary, and spare gases were 52.5, 13.75 and 2.75, respectively. Capillary temperature was 268.75°C. Normalized collision energies were 20% and 30%.

Data alignment and comparison between Nutlin-3b and Nutlin-3a treated groups were performed with either XCMS Online (https://xcmsonline.scripps.edu) or XCMS operated in R (https://bioconductor.org/packages/release/bioc/html/xcms.html). Mass accuracy, MS^2^, peak integration, and chromatography of all XCMS-identified lipids were verified via further examination of the raw data in Xcalibur (ThermoFisher). XCMS-generated area counts were used in data reporting, and data were normalized to total ion intensity between 150 *m/z* and 2000 *m/z* (TIC[150–2000]); all MS^2^ spectra matched known literature [40–43].

### Tumor cell preparation and flow cytometry

Pancreatic tumors were minced and dissociated into single cells by sequentially incubating them in 1) dissociation media for 1h at 37C with gentle agitation, 2) Trypsin 0.25% for 10 min at 37°C, and 3) ACK lysis buffer (ThermoFisher) for 2 min at room temperature to remove erythrocytes. Dissociation media was prepared freshly as follows: DMEM supplemented with 1 mg/mL Trypsin inhibitor (Gibco), 1 mg/mL Dispase II (Gibco), 1 mg/mL Collagenase IV (Gibco) and 0.025 mg/mL DNAse (Sigma). Final suspensions were passed through a 100 μm nylon mesh to remove aggregates. For labeling, cells were incubated on ice with Mouse Fc Block (BD Biosciences, 1/200) followed by antigen-specific antibodies, in FACS buffer (PBS with 1mM EDTA and 0.5% BSA). Cells were sorted at the Salk Institute FACS core facility on a BD Influx cell sorter. DAPI (Molecular Probes) was used to exclude dead cells. For labeling, the following antibodies were used: CD45-AF488, EPCAM-AF647, and PDGFRα-PE (Biolegend, 1/200).

### Cell immunostaining and confocal analyses

Cells were allowed to attach to glass coverslips overnight then treated with drugs for 72h. For visualization of lipid droplets, washed cells were fixed with 10% buffered formalin then stained with 1 μg/ml 4,4-difluoro-1,3,5,7,8-pentamethyl-4-bora-3a,4adiaza-s-indacene (BODIPY 493/503, Molecular Probes) for 1h at room temperature.

For immunostaining, cells were fixed with ice-cold 100% methanol, blocked with PBS containing 0.2% BSA and 0.05% Triton X-100, incubated with Ki-67 antibody (mouse monoclonal, BD 550609, 1/100) overnight at 4°C, and incubated with secondary fluorescent antibodies (Invitrogen) for 1h. Washed, stained cells were mounted using Vectastain mounting media containing DAPI (Vector Labs) and analyzed on an LSM780 confocal microscope (Zeiss).

### Tissue staining and quantification

Tumors were fixed overnight in zinc-containing, neutral-buffered formalin (Fisher Scientific), embedded in paraffin, cut in 5 μm sections, mounted, and stained. Sections were deparaffinized in xylenes, rehydrated in ethanol, and then washed in PBST and PBS. Endogenous peroxidase activity was blocked with a 1:50 solution of 30% H_2_O_2_:PBS followed by microwave antigen retrieval in 100 mM sodium citrate, pH 6.0. Sections were blocked with 1% bovine serum albumin and 5% normal goat serum in 10 mM Tris (pH 7.4); 100 mM MgCl_2_, and 0.5% Tween-20 for 1h at room temperature followed by an avidin/biotin blocking kit (Thermofisher) per the manufacturer’s instructions. The αSMA antibody (mouse monoclonal, sc-32251 Santa Cruz, 1/1000) was diluted in blocking solution and incubated over night. Slides were then washed, incubated in signal boost (Abcam) then developed with DAB substrate (Vectashield).

For Masson’s trichrome staining, slides were stained using a kit (Polysciences Inc) according to the manufacturer’s protocol. All slides were scanned and imaged on an Olympus VS-120 Virtual Slide Scanning microscope. For quantification of IHC or Masson’s Trichrome staining, at least eight 15x fields per scanned slide were scored using Inform 2.1 Advance Image Analysis software (PerkinElmer).

### RNA-seq library generation, High-throughput sequencing and analysis

RNA quality was assessed using the Agilent 2100 Bioanalyzer and RNA-Seq libraries were prepared using the TruSeq RNA Sample Preparation Kit v2 according to Illumina protocols. Multiplexed libraries were validated using the Agilent 2100 BioAnalyzer, normalized and pooled for sequencing. High-throughput sequencing was performed on the HiSeq 2500 system (Illumina). Image analysis and base calling were done with Illumina CASAVA-1.8.2. Sequenced reads were quality-tested using FASTQC and aligned to the hg19 or mm10 reference genomes using the STAR aligner version 2.4.0k. Mapping was carried out using default parameters (up to 10 mismatches per read, and up to 9 multi-mapping locations per read). The genome index was constructed using the gene annotation supplied with the hg19/mm10 Illumina iGenomes collection and sjdbOverhang value of 100. Raw gene expression was quantified across all gene exons (RNA-Seq) using the top-expressed isoform as proxy for gene expression, and differential gene expression was carried out using the edgeR package version 3.6.8 using duplicates to compute within-group dispersion and batch correction to account for the difference between cell lines. Pathway enrichment analysis was carried out using Ingenuity Pathway Analysis (QIAGEN), or Metascape [44] using default parameters. Heatmaps were generated from fragments per kilobase of exon per million mapped reads (fpkm) and either plotted as log2 of the fold change between two conditions or as z-normalized relative expression. RNA-Seq data reported in this paper have been deposited in the National Center for Biotechnology Information (NCBI) Sequence Read Archive (SRA) database, Accession # SRP118373

### Statistical analyses

Two-tailed Student’s t test, one-way ANOVA, and two-way ANOVA models were used to quantify significance. p values are represented as follows: *, p< 0.05; **, p< 0.01; ***, p< 0.001; #, p< 0.0001.

## Results

### Cancer-associated pancreatic stellate cells express functional p53

**Fig 1:**
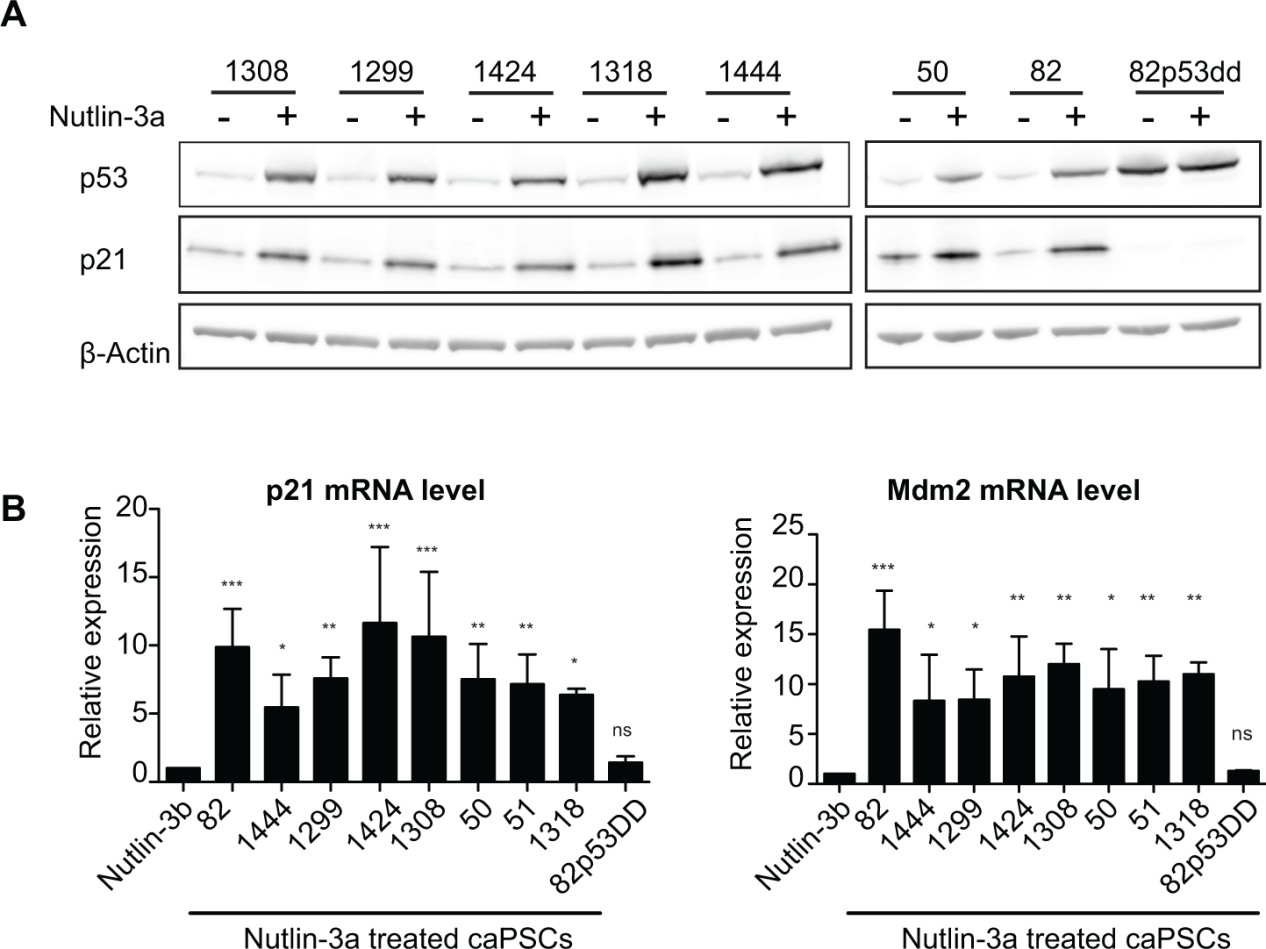
Cancer-associated pancreatic stellate cells express functional p53. (A) Immunoblot for p53 and p21 from primary caPSCs treated with Nutlin-3a (+) or its inactive enantiomer Nutlin-3b (-) for 48h. β-Actin serves as a loading control. (B) p21 and Mdm2 mRNA levels were quantified by real-time qPCR (RT-qPCR) in caPSCs treated with Nutlin-3a or Nutlin-3b for 48h. Values were normalized to Rplp0 mRNA levels and are represented as fold change relative to Nutlin-3b treated cells. Bars indicate mean +SD of at least 3 experiments. ***, p<0.001; **, p<0.01; *, p<0.05 by One-way ANOVA.

p53 mutations have been reported to occur in cancer-associated fibroblasts of colon and breast cancers [45], though this finding has been challenged [27,46]. To evaluate p53 functional status in cancer-associated pancreatic stellate cells (caPSCs), we isolated fibroblasts from 8 different patients with pancreatic ductal adenocarcinoma and treated the cells with Nutlin-3a or its inactive enantiomer Nutlin-3b. Treatment of caPSCs with Nutlin-3a induced p53 protein stabilization (Fig 1A) and increased expression of p53 target genes p21 and Mdm2 in all 8 samples (Fig 1A and 1B). This demonstrates that caPSCs express functional p53 and that the p53 transcriptional pathway is robustly activated by Nutlin-3a.

To control for potential p53-independent effects of Nutlin-3a, we introduced a dominant negative form of p53 (p53dd) into caPSCs derived from patient 82 (caPSC-82p53dd). p53dd is a p53 C-terminal fragment that forms transcriptionally inactive multimers with endogenous wild-type p53 protein, thereby blocking its transcriptional activity. As expected, p53dd increased wild-type p53 protein levels (Fig 1A), due to its ability to block Mdm2 transcription, which normally targets p53 for proteasomal degradation. Moreover, p53 transcriptional activity was inhibited by p53dd in cells treated with Nutlin-3a (Fig 1B), confirming that p53dd effectively inactivates p53 and prevents Nutlin-3a from functioning.

### p53 reprograms human caPSCs towards quiescence

**Fig 2:**
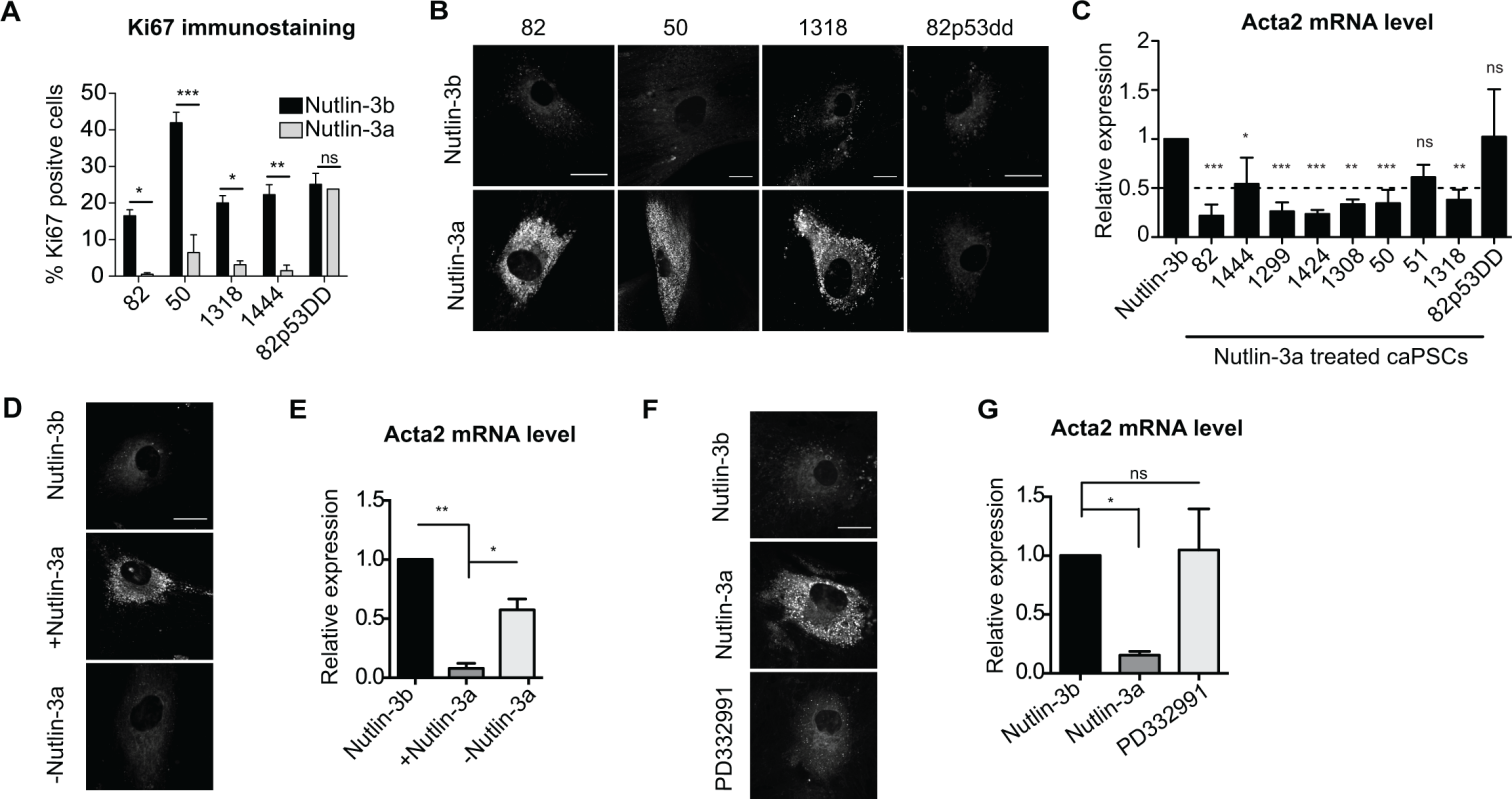
p53 reprograms human caPSCs towards quiescence. caPSCs were treated with Nutlin-3a, Nutlin-3b (control) or PD332991 for 48h (C, G) or 72h (A,B, F). (A) Cells were immunostained for Ki67 and nuclei were stained with DAPI. Bar graph represents the percentage of nuclei positive for the Ki67 antigen. At least 100 cells per condition were counted. Values are plotted as mean +SD of at least 2 experiments. ***, p<0.001; **, p<0.01; *, p<0.05 by two-way ANOVA. (B) Representative images of caPSCs stained with BODIPY 493/503 for detection of neutral lipids. (C) Acta2 mRNA levels were quantified by RT-qPCR. Values were normalized to Rplp0 mRNA levels and are represented as fold change relative to Nutlin-3b treated cells. Bars indicate mean +SD of at least 3 experiments. ***, p<0.001; **, p<0.01; *, p<0.05 by one-way ANOVA. (D-E) caPSCs were treated with Nutlin-3a or Nutlin-3b for 72h. Nutlin-3a was removed from treated caPSCs and cells were cultured for an additional 72h with (+) or without (-) Nutlin-3a. (D) Representative images of cells stained with BODIPY 493/503 (E) Acta2 mRNA levels were quantified and represented as described in C. (F) Representative images of cells stained with BODIPY 493/503. (G) Acta2 mRNA levels were quantified and represented as described in C. Scale bars, 25 μM.

We next tested the ability of Nutlin-3a to reprogram activated caPSCs. caPSCs treated with Nutlin-3a or Nutlin-3b (control) for 72h were stained with the proliferation marker Ki67. As expected, the number of proliferating cells was greatly reduced upon Nutlin-3a treatment in caPSCs, but not in the caPSC-82p53dd control (Fig 2A). Interestingly, in all 8 independent caPSC lines tested, Nutlin-3a induced accumulation of cytoplasmic lipid droplets (Fig 2B and S1A Fig) and downregulation of αSMA expression (Acta2, Fig 2C), whereas no changes were observed in caPSC-82p53dd (Fig 2B and 2C). These data indicate that Nutlin-3a induces phenotypic changes associated with quiescence and that these changes are strictly mediated by p53.

p53 can induce reversible (quiescence) or irreversible (senescence) cell cycle arrest. To distinguish between these two phenotypes, Nutlin-3a was removed from previously treated caPSCs, and the cells were then cultured for an additional 3 days with (+) or without (-) Nutlin-3a. Removing Nutlin-3a for 3 days reduced p53 transcriptional activity to its basal level, as determined by p21 expression (S1B Fig). Furthermore, removal of Nutlin-3a led to an increase in proliferation (S1C Fig), loss of lipid droplets (Fig 2D) and upregulation of αSMA expression (Fig 2E), demonstrating that the effects of p53 activation are reversible.

To determine whether the caPSC quiescence-like phenotype is a manifestation solely of cell cycle arrest, we assessed the effects of a CDK4/6 inhibitor on caPSC activation. Interaction of CDK4/6 with cyclin D1 results in retinoblastoma protein (Rb) phosphorylation and, consequently, release of transcription factors that allow cell cycle progression to S phase [47]. Treatment of caPSC-82 and caPSC-82p53dd with the CDK4/6 inhibitor PD332991 induced Rb dephosphorylation (S1D Fig) and cell cycle arrest (S1E Fig), showing that PD332991 induces cell cycle arrest independently of p53 activity. Nonetheless, CDK4/6 inhibition did not induce accumulation of lipid droplets (Fig 2F) or downregulation of αSMA (Fig 2G). Taken together, these data support the idea that growth arrest alone is not sufficient to reprogram caPSCs towards quiescence, and that downstream effectors of p53 are involved.

### p53 transcriptionally regulates the PSC activation network

**Fig 3:**
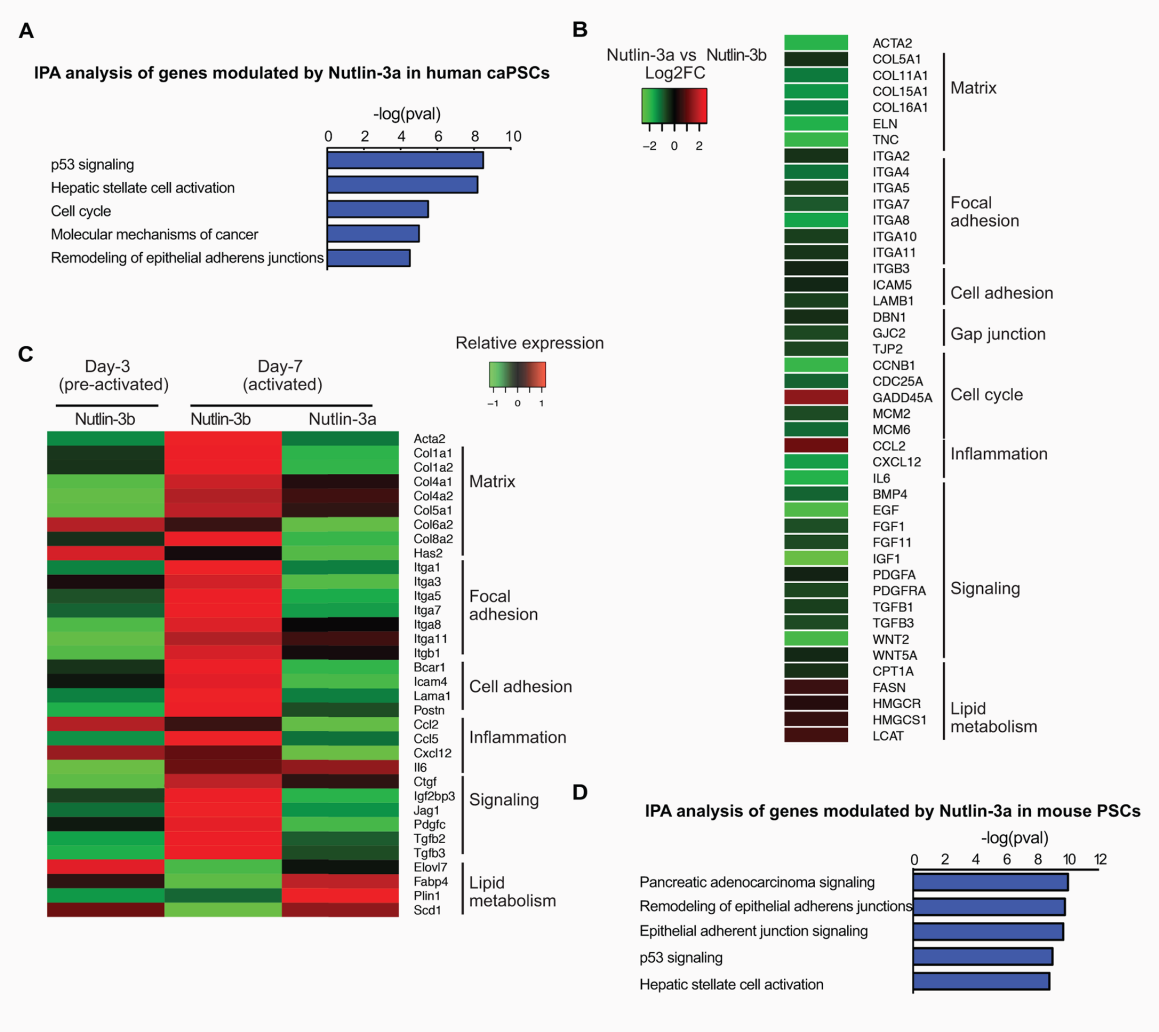
p53 transcriptionally regulates the PSC activation network. (A-B) Four primary human caPSCs were treated for 48h with Nutlin-3a or Nutlin-3b (control) and analyzed by RNA-seq. (A) Ingenuity Pathway Analysis (IPA) was performed on p53 downregulated and upregulated genes from RNA-seq analysis (fold change >1.5 or <0.67, adjusted p <0.05). The 5 most significant canonical pathways are shown and–log(pval) are indicated. (B) Heatmap representing selected genes from the RNA-seq analysis. Data are represented as log2 fold change, Nutlin-3a versus Nutlin-3b. (C-D) Primary mouse PSCs isolated from pancreata of wild-type C57B6/J mice were treated with Nutlin-3a or Nutlin-3b and harvested on day 3 (pre-activated) or on day 7 (activated) of culture. (C) Heatmap shows the relative abundance of selected genes from the RNA-seq analysis of Nutlin-3b treated mPSC (day 3 and 7) and Nutlin-3a treated mPSC (day 7). (D) IPA analysis was performed on p53 downregulated and upregulated genes at day 7 (fold change>1.5 or <0.67, adjpval<0.05). The 5 most significant canonical pathways are shown and -log(pval) are indicated.

To assess the genome-wide effects of p53 activation, we analyzed the transcriptome of four human caPSC lines cultured with Nutlin-3a or its inactive enantiomer for 48h. p53 activation induced upregulation of 1331 genes and downregulation of 1473 genes (adjusted p<0.05, fold-change>1.5 or <0.67). Ingenuity Pathway Analysis (IPA) of p53-modulated genes revealed profound effects on p53 signaling, stellate cell activation, cell cycle, and molecular mechanisms of cancer (Fig 3A). caPSCs are characterized by gene signatures that include ECM components, cell adhesion molecules, inflammatory mediators, signaling molecules and genes involved in lipid metabolism [14]. We found that p53 inhibits the activation gene signature of caPSCs (Fig 3B). Indeed, p53 activation resulted in decreased expression of potential tumor-supporting genes such as cytokines/chemokines (IL-6, CXCL12), growth factors (PDGF, FGF1, IGF1), signaling molecules (TGFβ1, WNT5) and ECM components (Collagens, Elastin). p53 also modulates genes involved in cell cycle and lipid metabolism (Fig 3B), consistent with the decreased proliferation and increased lipid droplet accumulation observed upon Nutlin-3a treatment (Fig 2A and 2B). This analysis suggests that p53 modulates caPSC activation through transcriptional regulation of the PSC activation network.

To determine if p53 can prevent activation of PSCs, quiescent mouse PSCs (mPSCs) were isolated from wild-type, healthy murine pancreata and cultured in monolayer to induce activation [8]. mPSC activation was characterized by a loss of cytoplasmic lipid droplets (S2A Fig) and an increase in αSMA and Col1a1 expression (S2B Fig). Nutlin-3a treatment of mPSCs prevented this increase in αSMA and Col1a1 expression (S2B Fig). Conversely, Nutlin-3a treatment of mPSCs isolated from p53 knock-out mice did not induce expression of p53 target genes (p21 and Mdm2) and had no effect on either αSMA or Col1a1 expression (S2C and S2D Fig). To determine the genome-wide effects of p53 activation in mPSCs, we performed RNA-seq analysis on pre-activated (3-day culture) and activated (7-day culture) mPSCs treated with Nutlin-3a or its inactive enantiomer. Consistent with previous studies [14], mPSC activation resulted in increased expression of matrix components, cell adhesion molecules, signaling molecules, growth factors and cytokines (Fig 3C: Nutlin-3b day-3 vs day-7). Importantly, p53 activation prevented upregulation of these genes (Fig 3C, day 7 Nutlin-3b vs Nutlin-3a), suggesting that p53 activation prevents in vitro mPSC activation. Additionally, IPA analysis of p53-modulated genes demonstrates that p53 controls expression of genes involved in pancreatic cancer signaling, remodeling of adherens junctions, and stellate cell activation (Fig 3D). Taken together, these data support the hypothesis that p53 pathway activation induces PSC quiescence through transcriptional regulation of the PSC activation gene network.

### p53-induced lipid droplet accumulation is associated with increased triacylglycerols and cholesterol esters in fibroblasts

**Fig 4:**
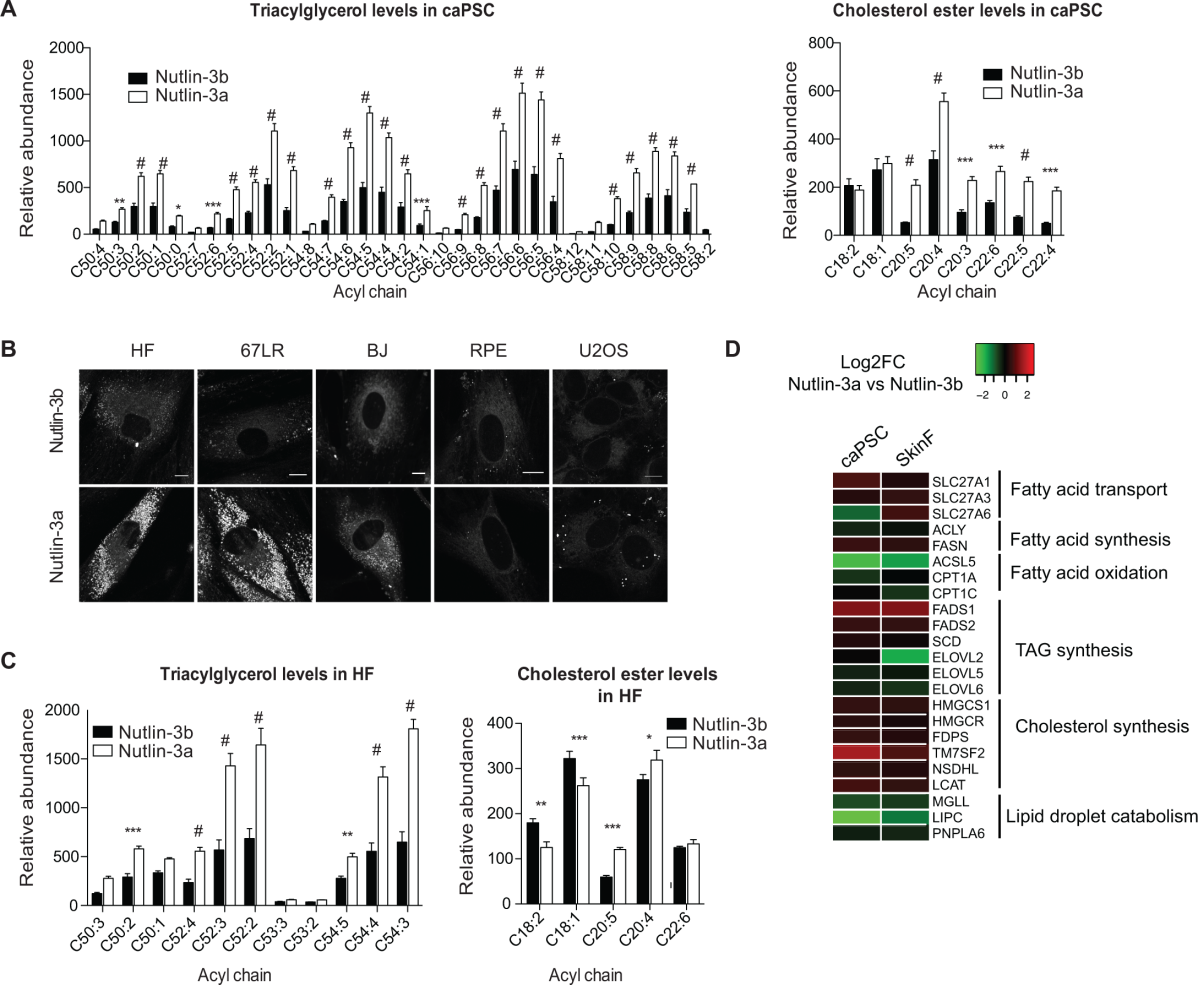
p53-induced lipid droplet accumulation is associated with an increase in triacylglycerols and cholesterol esters. (A) Relative abundance of selected lipids from mass spectrometry-based lipidomics analysis of caPSC-82 treated for 72h with Nutlin-3a or Nutlin-3b (control). Bar graphs represent mean +SD of triplicates. #, p<0.0001; ***, p<0.001; **, p<0.01; *, p<0.05 by two-way ANOVA. (B) Representative images of cells treated with Nutlin-3a or Nutlin-3b for 72h and stained with BODIPY 493/503. Scale bar, 10 μM. (C) Relative abundance of selected lipids from mass spectrometry-based lipidomic analysis of the skin fibroblast line HF treated for 72h with Nutlin-3a or Nutlin-3b. Data are represented as in A. (D) Heatmap representing selected genes from the RNA-seq analysis of caPSCs and skin fibroblasts (SkinF). Data are represented as log2 fold change, Nutlin-3a versus Nutlin-3b.

Lipid droplets are dynamic intracellular bodies composed of a central core of neutral lipids, mainly comprised of triacylglycerols (TAGs) and sterol esters, shielded by a phospholipid monolayer membrane [48]. Nutlin3a-treated caPSCs are characterized by an abundance of cytoplasmic lipid droplets (Fig 2B). To identify changes in cellular lipid species associated with p53 activation, we conducted a mass spectrometry-based lipidomic analysis [49] of human caPSC-82 treated with Nutlin-3a or its inactive enantiomer Nutlin-3b. Interestingly, p53 activation leads to an increase in many TAGs and cholesterol esters (Fig 4A), whereas no increase was observed in diacylglycerol (DAG) or monoacylglycerol (MAG) levels (S3A Fig).

To determine if p53-induced lipid droplet accumulation is a general function of p53, we assessed the effect of p53 activation on lipid droplet accumulation in three different primary skin fibroblast lines (BJ, HF, 67LR), retinal epithelial cells (RPE), and an osteosarcoma cell line encoding wild type p53 (U2OS). These cells were treated with Nutlin-3a or control. Although all cell lines showed an increase in p53 protein expression and transcriptional activity upon Nutlin-3a treatment (S3B and S3C Fig), lipid droplet accumulation was only observed in the three fibroblast cultures (Fig 4B). Similar to caPSCs, CDK4/6 inhibition in skin fibroblasts inhibited Rb phosphorylation but did not induce lipid droplet accumulation (S3D and S3E Fig). Moreover, lipidomic analysis of the skin fibroblast line, HF, treated with Nutlin-3a showed that p53 activation increased levels of several TAG species and, to a lower extent, levels of cholesterol esters species, while levels of DAG were slightly reduced (Fig 4C and S3F Fig). These data indicate that p53 activation results in accumulation of lipid droplets and increased levels of TAGs and cholesterol esters in fibrolasts isolated from skin and pancreatic tissues.

To identify potential p53 target genes that control lipid pathways, we performed RNA-seq analysis on two skin fibroblast lines treated with either Nutlin-3a or Nutlin-3b for 48h. 364/512 genes upregulated by Nutlin-3a in the skin fibroblasts were also increased in the caPSCs and 269/404 of downregulated genes were also reduced in caPSCs in comparison to Nutlin-3b treated cells (adjpval<0.05, FC>2 or <0.5). We used Metascape [44] to test for over-represented pathways and GO terms using genes upregulated or downregulated in both caPSCs and skin fibroblasts. As expected, Metascape-enriched gene clusters showed significant upregulation of p53 pathway genes and a significant downregulation of cell cycle related genes (S3G Fig). Interestingly, upregulated genes were enriched for the lipid transport cluster, suggesting that p53 transcriptionally regulates genes involved in lipid droplet formation. Moreover, we found that p53 activation modulates the expression of genes involved in fatty acid uptake and metabolism, lipid synthesis, as well as lipid droplet catabolism (Fig 4D). These transcriptional changes could potentially affect the levels of TAGs and cholesterol esters in cells and result in lipid droplet accumulation. Taken together, these data indicate a general mechanism for p53-induced lipid droplet accumulation in fibroblasts, which is controlled, at least in part, through transcriptional regulation.

### Stromal p53 activation reduces pancreatic desmoplasia in vivo

**Fig 5:**
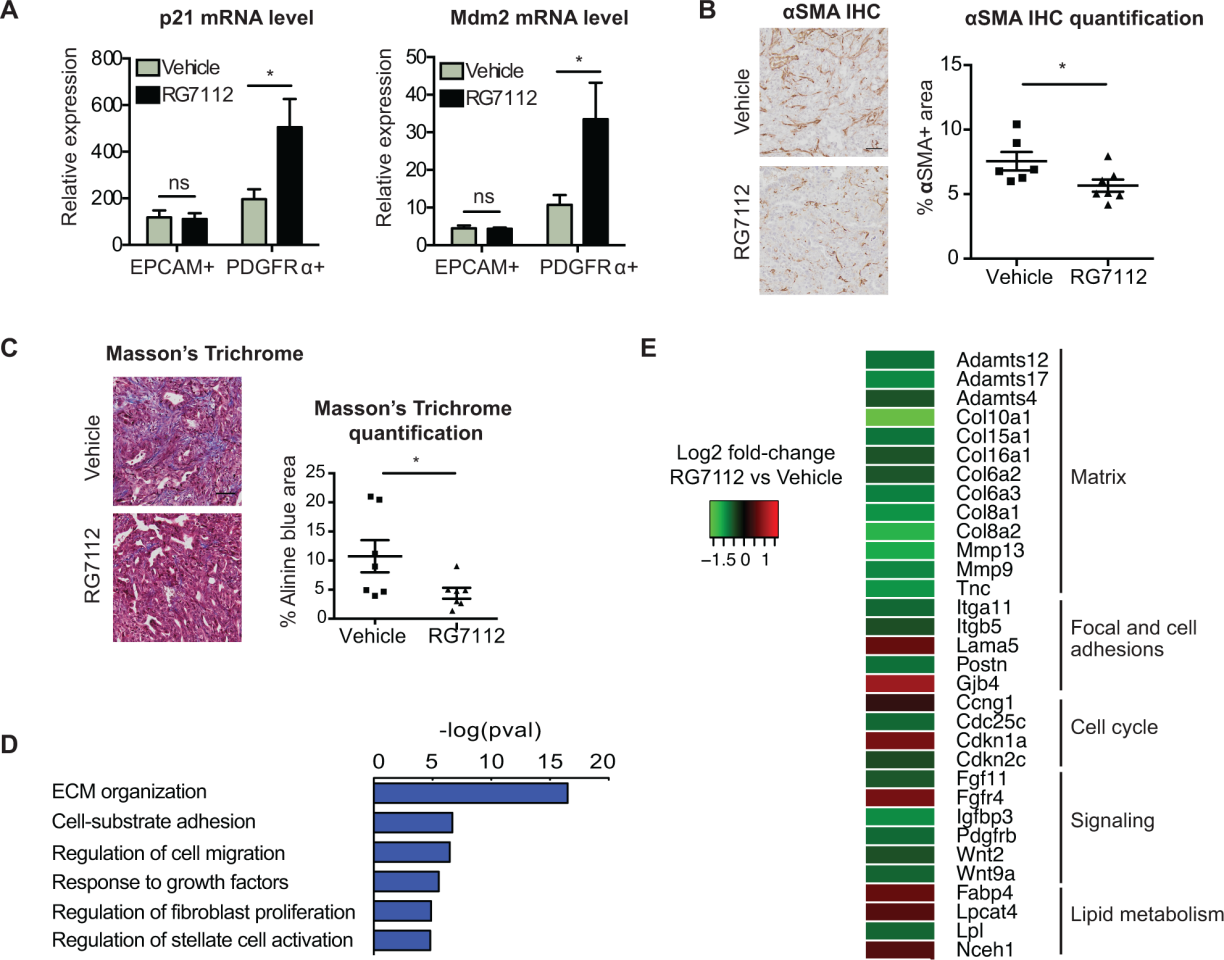
Stromal p53 activation reverses caPSC activation and reduces pancreatic desmoplasia in vivo. (A) Tumor bearing mice were treated with vehicle or RG7112 (200 mg/kg) and were sacrificed. Epithelial cells (EPCAM+) and fibroblasts (PDGFRα+) were isolated from dissociated tumors by FACS. p21 and Mdm2 mRNA levels were quantified by RT-qPCR and normalized to Rplp0 mRNA. Bars represent mean +SD of 3 different mice. *, p<0.05 by two-way ANOVA. (B-C) Mice transplanted with KPC cells were treated for 15 days with Vehicle or RG7112 (200 mg/kg) starting day 8 post-transplantation. Tumors were harvested and fixed in formalin. FFPE sections were subjected to (B) IHC using an αSMA antibody and (C) Masson’s Trichrome staining. Representative images are shown on the left and quantification on the right. At least eight 15X fields were quantified using Inform 2.1 software and mean values for each tumor are plotted on the graph. *, p<0.05 by Student’s test. Scale bar, 50 μM. (D) Metascape analysis was performed on RG7112 downregulated and upregulated genes (fold change >1.4 or <0.7, adjusted p<0.05). Six of the twenty most significant canonical pathways are shown and -log(pval) are indicated. (E) Heatmap representing selected genes from the RNA-seq analysis. Data are represented as log2 fold change, RG7112 versus Vehicle.

These promising *in vitro* results led us to explore the impact of stromal p53 activation *in vivo*. We used the clinical form of Nutlin-3a (RG7112) from Roche (Basel, Switzerland) for these studies since it is a more potent p53 activator. *In vitro*, mPSCs treated with RG7112 showed an increase in p53 transcriptional activity similar to Nutlin-3a treated cells (S4A Fig). *In vivo*, RG7112 treatment of wild-type mice (75 mg/kg and 200 mg/kg) induced an increase in p53 and p21 protein levels in the pancreas, indicating that the drug is infiltrating the intended target organ (S4B Fig). We then assessed whether RG7112 activates p53 in the stroma of pancreatic tumor-bearing mice. For these experiments, we used an orthotopic allograft model in which pancreatic tumor cells are transplanted into the pancreata of immunocompetent mice. Cells derived from *KRas*^*LSL-G12D/+*^*;Trp53*^*LSL-R172H/+*^*;Pdx1-Cre* mice (KPC cells) were used for transplantation. Treatment of KPC cells *in vitro* with RG7112 failed to result in p53 activation (S4A and S4C Fig), suggesting that this KPC cell line has lost its remaining wild-type p53 allele and is unaffected by RG7112 treatment. To confirm that we can efficiently isolate cancer and fibroblast cell populations, we used Fluorescence-Activated Cell Sorting (FACS) to isolate cancer cells (CD45-EPCAM+) and caPSCs (CD45-EPCAM-PDGFRα+) from KPC allograft tumors. PDGFRα allows isolation of both normal and cancer-associated fibroblasts, including but not restricted to αSMA+ activated fibrolasts [50]. PDGFRα+ sorted cells were characterized by a fibroblastic morphology in culture (S4D Fig), low expression of epithelial markers (Krt19 and Cdh1) and high expression of fibroblast markers (αSMA and Col1a1) as compared to EPCAM+ cells (S4E Fig). We used this sorting approach to determine whether RG7112 activates p53 in fibroblasts within allograft tumors. Tumor-bearing mice were treated with a single dose of RG7112 (200 mg/kg) or vehicle; 24 hours later PDGFRα+ and EPCAM+ cells were isolated from tumors and expression of p53 target genes were assessed by RT-qPCR. PDGFRα+ cells isolated from RG7112-treated mice showed an upregulation of p53 target genes as compared to cells isolated from vehicle-treated tumors (Fig 5A). In contrast, no changes in p53-specific target genes were observed in isolated cancer cells (EPCAM+) (Fig 5A). These data show that RG7112 effectively activates p53 in caPSCs and can be used to target stromal p53 in PDAC.

We then examined the effects of continuous p53 activation on the tumor stroma. Mice were transplanted with KPC cells then treated daily for 15 days with either RG7112 or Vehicle starting day 8 post-transplantation. Importantly, we found that RG7112 reduced the number of activated caPSCs, as marked by αSMA expression (Fig 5B). This change was accompanied by a decrease in collagen deposition, measured by Masson’s trichrome staining (Fig 5C). To further decipher the effects of RG7112 on stromal cells, PDGFRα+ cells were isolated from RG7112- and Vehicle-treated tumors by FACS then analyzed by RNA-seq. RG7112 induced gene expression changes in 228 genes (adjusted p<0.05, fold-change>1.4 or <0.7). Metascape analysis of RG7112-modulated genes revealed effects on extracellular matrix organization, cell adhesion, cell migration, response to growth factors, proliferation and stellate cell activation (Fig 5D). This analysis suggests that p53 transcriptionally regulates caPSC activation *in vivo*. We found that RG7112 decreases expression of ECM components (Collagens, Tenascin) and ECM remodeling proteins (MMPs, Adamts) in PDGFRα+ cells, compared to vehicle (Fig 5E). These effects are consistent with the observed decrease in collagen deposition (Fig 5C). Consistent with our *in vitro* data (Fig 3B and 3C), RG7112 modulates expression of genes involved in cell/focal adhesion (Itgb5, Lama5), signaling (Fgf11, Igfbp3, Wnt2), cell cycle (Cdkn1a, Cdc25a) and lipid metabolism (Fabp4, Lpl) (Fig 5E). All together our data indicate that RG7112 is able to reverse caPSC activation in vivo, resulting in decreased expression of tumor-supportive genes and reduced desmoplasia.

## Discussion

Pancreatic cancer is characterized by a supportive microenvironment, which promotes tumor growth and invasion and actively participates in resistance to conventional chemotherapeutics [51]. Targeting the stroma of pancreatic cancer has, therefore, emerged as a potential therapeutic strategy to improve the dismal prognosis associated with this disease. Various strategies have been examined, such as targeting PSCs, inhibiting ECM deposition, suppressing angiogenesis, and re-engineering the host immune response [52]. Interestingly, induction of quiescence in caPSCs with all-trans retinoic acid (ATRA) has been shown to reduce cancer cell proliferation and invasion and to increase apoptosis [53]. Moreover, vitamin D receptor (VDR) activation reverts caPSCs activation, resulting in reduced inflammation and fibrosis, inhibition of tumor growth, and increased survival in preclinical models [14]. We show here that the transcription factor p53 is also a regulator of caPSC activation. Activation of p53 reprograms caPSCs towards quiescence by reducing proliferation, decreasing αSMA expression, and inducing accumulation of cytoplasmic lipid droplets. TGFβ and platelet derived growth factor (PDGF) are potent activators of caPSCs. TGFβ stimulates the synthesis and secretion of ECM proteins, while PDGF induces proliferation and chemotaxis in caPSCs [6]. Interestingly, we found that p53 activation downregulates expression of both factors, which may reduce caPSC activation via autocrine pathways. p53 activation also triggers transcriptional changes in caPSCs, which may limit their tumor supportive functions. For example, p53 activation downregulates ECM components such as collagens [10,13], the mitogenic epithelial factors EGF and FGF1 [54], chemokine IL6 [55] and cytokine CXCL12, which is a mediator of the T cell response and cancer cell invasion [56,57].

Our work reveals that p53 is a key regulator of caPSC activation and quiescence. These data are consistent with previous studies showing that loss of stromal p53 promotes cancer-associated fibroblast activation in hepatocellular and squamous cell carcinomas [25,29]. If stromal p53 indeed controls fibroblast activation and inhibits tumor supportive functions, tumor cells may respond by evolving mechanisms to overcome stromal p53 effects. p53 mutations were reported in laser-capture microdissected or cultured fibroblasts isolated from the stroma of breast and colon cancers [45,58,59]. However, these studies have been challenged, and more recent analyses reveal that the p53 mutations purported to be in the stroma most likely result from technical challenges and subsequent contamination by epithelial cancer cells [27,46]. Our data clearly show that caPSCs isolated from pancreatic cancer patients express functional p53 and, therefore, support the idea that stromal p53 is not mutated during tumorigenesis. Alternatively, tumor cells could overcome the effects of stromal p53 through paracrine mechanisms. Indeed, it has been reported that conditioned-media from non-small cell lung cancer cells suppresses stromal p53 induction upon genotoxic stresses [28]. Moreover, p53 expression was found to be lower in squamous cell carcinoma-associated fibroblasts as compared to normal dermal fibroblasts, and FGF-2 was identified as a paracrine factor responsible for this downmodulation [29]. Pancreatic cancer cells secrete many growth factors and cytokines able to induce PSC activation [7], and it remains to be determined whether these factors also modulate stromal p53 activity.

One manifestation of PSC quiescence is the accumulation of lipid droplets. Conversely, PSC activation is typically characterized by loss of lipid droplets. Lipid droplets are specialized, dynamic organelles that store energy in the form of neutral lipids such as triacylglycerols (TAGs) and sterol esters [60]. Lipid droplets are formed from the endoplasmic reticulum, where most cellular lipids are synthetized. Cells metabolize neutral lipids when energy is needed in the form of fatty acids and/or for membrane synthesis. Here, we show for the first time that non-genotoxic activation of p53 by Nutlin-3a induces lipid droplet formation and TAG and cholesterol ester accumulation in fibroblasts undergoing quiescence. Interestingly, TAGs and lipid droplets have also been shown to accumulate in fibroblasts undergoing replicative senescence [61] and during early-apoptosis in etoposide-treated lymphoma cells [62], although a role for p53 has not been investigated in these contexts.

In general, p53 is thought to function as a negative regulator of lipid synthesis by activating fatty acid oxidation and by inhibiting fatty acid synthesis [63–65]. However, in our study, p53 activation resulted in an increase in levels of multiple lipid species and lipid droplets. It is likely that p53 function in lipid metabolism varies depending on cell type and context. For example, it has been shown in a variety of cancer cell lines that p53 upregulates expression of the carnitine pamitoyltransferase CPT1C, which is involved in the transport of fatty acids into the mitochondria for fatty acid oxidation [66]. However, we found that p53 activation downregulates CPT1A and CPT1C in caPSCs and skin fibroblasts, respectively, which could result in decreased fatty acid oxidation. Consistent with this, our preliminary data suggest that fatty acid oxidation is reduced upon p53 activation in fibroblasts. p53 has also been demonstrated to repress the expression of the sterol regulatory element-binding protein 1 (SREBP1), a key transcription factor required for the synthesis of cholesterol, fatty acids, triacylglycerols and phospholipids [64]. However, our RNA-seq data indicate that SREBP1 is unaffected by p53 activation, whereas SREBP2 is upregulated in the caPSC. Again, this suggests cell context-dependent effects of p53 activation and demonstrates how further analysis of lipid storage in PSCs from other organs and disease states is warranted.

Lineage specific deletion of p53 in hepatic stellate cells enhances liver fibrosis in mice treated with the fibrosis-inducing agent carbon tetrachloride (CCl_4_) and is associated with an increase in fibroblast proliferation and ECM deposition [25]. Here, we show that stromal p53 activation decreases expression of ECM proteins and remodeling proteins in fibroblasts isolated from tumors and decreases collagen deposition in an allograft model of pancreatic cancer. While p53 activation impacted disease progression in both models, these effects were modest. This may be due to the fact that the disease state in both of these models is characterized by a relative paucity of fibrosis. In particular, desmoplasia in allograft models is subtle compared to genetically engineered mouse models (GEMMs) or human pancreatic cancer [67]. Further investigation using GEMMs is, therefore, warranted. There are several tactics that could be taken to better understand the effects of stromal p53 activation and its clinical relevance. For example, to better assess the effects of stromal p53 on pancreatic tumorigenesis, the *FSF-Kras*^*G12D/+*^*; Pdx1-FLP* model could be crossed to *p53*^*floxed/floxed*^ and *GFAP-Cre* (for PSCs) or *Col1a1-CreERT2* (for all fibroblasts) to determine if a lack of wild type p53 in the stroma is pro- or anti-tumorigenic. To better assess the clinical relevance of p53 activation as a therapeutic, the *LSL-Kras*^*G12D*^*;p53*^*R172H/R172H*^*;Pdx1-Cre* model, which has an abundance of stroma, could be treated with RG1172 alone or in combination with gemcitabine to determine clinical benefit.

Similar to the vitamin D analog Calcipotriol, we found that the p53 activator Nutlin-3a blocks murine PSC activation through transcriptional regulation. Interestingly, an increasing body of evidence points to crosstalk between p53 and Vitamin-D signaling in various contexts [68]. To compare the effects of VDR and p53 in mPSC, we performed a Metacore analysis of the RNA sequencing data shown here and from Sherman *et al*. [14]. Pathway enrichment analysis reveals that genes commonly regulated by Calcipotriol and Nutlin-3a are enriched for cell cycle, cytoskeleton remodeling, immune response, cell adhesion and ECM remodeling pathways. This analysis also reveals that many genes within these pathways are uniquely regulated by VDR or p53. These observations suggest that p53 and VDR control mPSC activation through both common and distinct pathways. Interestingly, it was shown that a Vitamin-D analog accelerates Nutlin3a-induced apoptosis in acute myeloid leukemic cells [69]. These data imply that Nutlin-3a and Calcipotriol may cooperate, and that co-treatment of mPSC may serve to further block mPSC activation and, therefore, tumorigenesis.

Combination treatment with the standard of care chemotherapeutic, Gemcitabine, and stromal remodeling agents has been reported to decrease desmoplasia and improve survival in mice as compared to Gemcitabine monotherapy [13,14]. Gemcitabine is a nucleoside analog that incorporates into replicating DNA and blocks DNA synthesis, which eventually results in cell death. Resting cells do not undergo large-scale DNA replication and are therefore affected little by such treatment. Pharmacologic activation of p53 with Nutlin-3a has been shown to protect wild-type p53 expressing cells against treatment with nucleoside analogues [70]. Therefore, we suggest that pre-treatment of tumor-bearing mice with p53 agonists may; 1) decrease desmoplasia and thereby improve tumor accessibility and efficacy of Gemcitabine, and 2) protect normal cells from the genotoxic effects of Gemcitabine. This hypothesis will be important to test in mouse GEMMs with PDAC-specific p53 mutations, wild type p53 in the stroma, and sufficient desmoplasia to provide a realistic mimic for human PDAC.

## Supporting information

S1 Fig PD332991 induces Rb dephosphorylation and cell cycle arrest independently of p53 (related to Fig 2). (A) Representative images of caPSCs stained with BODIPY 493/503. (B-C) Cells were treated as described in Fig 2. (B) p21 mRNA level was quantified by RT-qPCR. (C) Bar graph indicates the percentage of nuclei positive for the antigen Ki67. (D-E) caPSCs were treated for 72h with Nutlin-3b, Nutlin-3a or PD332991 (D) Immunoblot of the indicated proteins. β-Actin serves as a loading control. (E) Bar graph indicates the percentage of cells positive for the antigen Ki67. Bar graphs are represented as in Fig 2. Scale bar, 25 μM.
(TIF)

S2 Fig Nutlin-3a does not block mPSC activation in p53 knock-out cells (related to Fig 3). (A) Primary mouse PSCs were isolated from pancreata of wild-type C57B6/J mice and stained with BODIPY 493/503 on days 1, 4 and 7 of culture. Representative images are shown. Scale bar, 10 μM. (B-D) Primary mouse PSCs isolated from pancreata of p53 wild-type (p53^+/+^) or p53 knock-out (p53^-/-^) mice were treated with Nutlin-3a or Nutlin-3b and harvested on days 3 and 7 of culture. mRNA levels of the indicated genes were assessed by RT-qPCR and normalized to Rplp0 mRNA. Bars represent mean + SEM of 5 experiments. ***, p<0.001; *, p<0.05 by two-way ANOVA.
(TIF)

S3 Fig p53 activation does not induce an increase in diacylglycerols and monoacylglycerols (related to Fig 4). (A) Relative abundance of selected lipids from mass spectrometry-based lipidomic analysis of caPSC-82 treated for 72h with Nutlin-3a or Nutlin-3b, represented as in Fig 4. (B-C) Cells were treated for 48h with Nutlin-3b (-) or Nutlin-3a (+). (B) Immunoblot for p53, β-Actin serves as a loading control. (C) p21 and Mdm2 mRNA levels were quantified by RT-qPCR. Values were normalized to Rplp0 mRNA levels and are represented as fold change relative to Nutlin-3b treated cells. Bars indicate mean +SD of at least 2 experiments. ***, p<0.001; **, p<0.01; *, p<0.05 by one-way ANOVA. (D-E) The skin fibroblast lines HF and 67LR were treated for 72h with Nutlin-3b (Nut3b), Nutlin-3a (Nut3a) or PD332991 (PD). (D) Immunoblot for the indicated proteins. β-Actin serves as a loading control; (E) Representative images of cells stained with BODIPY 493/503. Scale bar, 10 μM. (F) Relative abundance of selected lipids from mass spectrometry-based lipidomic analysis of the skin fibroblast line HF treated for 72h with Nutlin-3a or Nutlin-3b, represented as in Fig 4. (G) Genes regulated in both caPSCs and skin fibroblasts (Nutlin-3a vs Nutlin-3b, adjusted p<0.05, fold-change>2 or < 0.5) were analyzed with Metascape. The 20 most significant canonical pathways are shown for p53 upregulated genes (left) and downregulated genes (right).
(TIF)

S4 Fig RG7112 activates p53 in vitro and in vivo (related to Fig 5). (A) mPSC and KPC cells were treated with Nutlin-3a, RG7112 or control compounds (inactive enantiomers) for 48h. Mdm2 and p21 mRNA levels were assessed by RT-qPCR. Values were normalized to Rplp0 mRNA levels and are represented as fold change relative to the control. Bars indicate mean +SD of 2 experiments. **, p<0.01; *, p<0.05 by one-way ANOVA. (B) Wild-type C57B6/J mice were treated with RG7112 (75 or 200 mg/kg) or vehicle and pancreata were harvested 24h later. p53 and p21 protein levels were analyzed by Western-blot. β-Tubulin serves as a loading control. (C) Immunoblot for p53 from KPC cells treated for 24h and 48h with Nutlin-3a (+) or Nutlin-3b (-). β-Tubulin serves as a loading control. (D-E) Tumors were harvested from transplanted mice and dissociated. EPCAM+ and PDGFRα+ cells were isolated as described in Fig 5. (D) Representative pictures of day 3 of culture. (E) mRNA levels of the indicated genes were assessed by RT-qPCR and normalized to Rplp0 mRNA levels. Mean +SEM for at least 3 mice were plotted. ***, p<0.001; **, p<0.01; *, p<0.05 by Student’s test.
(TIF)

S5 Fig Uncropped and un-altered blot images used to make the figures. (TIF)

S1 Table Primers used in this study (related to experimental procedures). (PDF)
