# Supplementary figures and images for: Reprogramming pancreatic stellate cells via p53 activation: A putative target for pancreatic cancer therapy
Source: PLoS One. 2017 Dec 6;12(12):e0189051. doi: 10.1371/journal.pone.0189051 (PMC5718507; doi:10.1371/journal.pone.0189051)

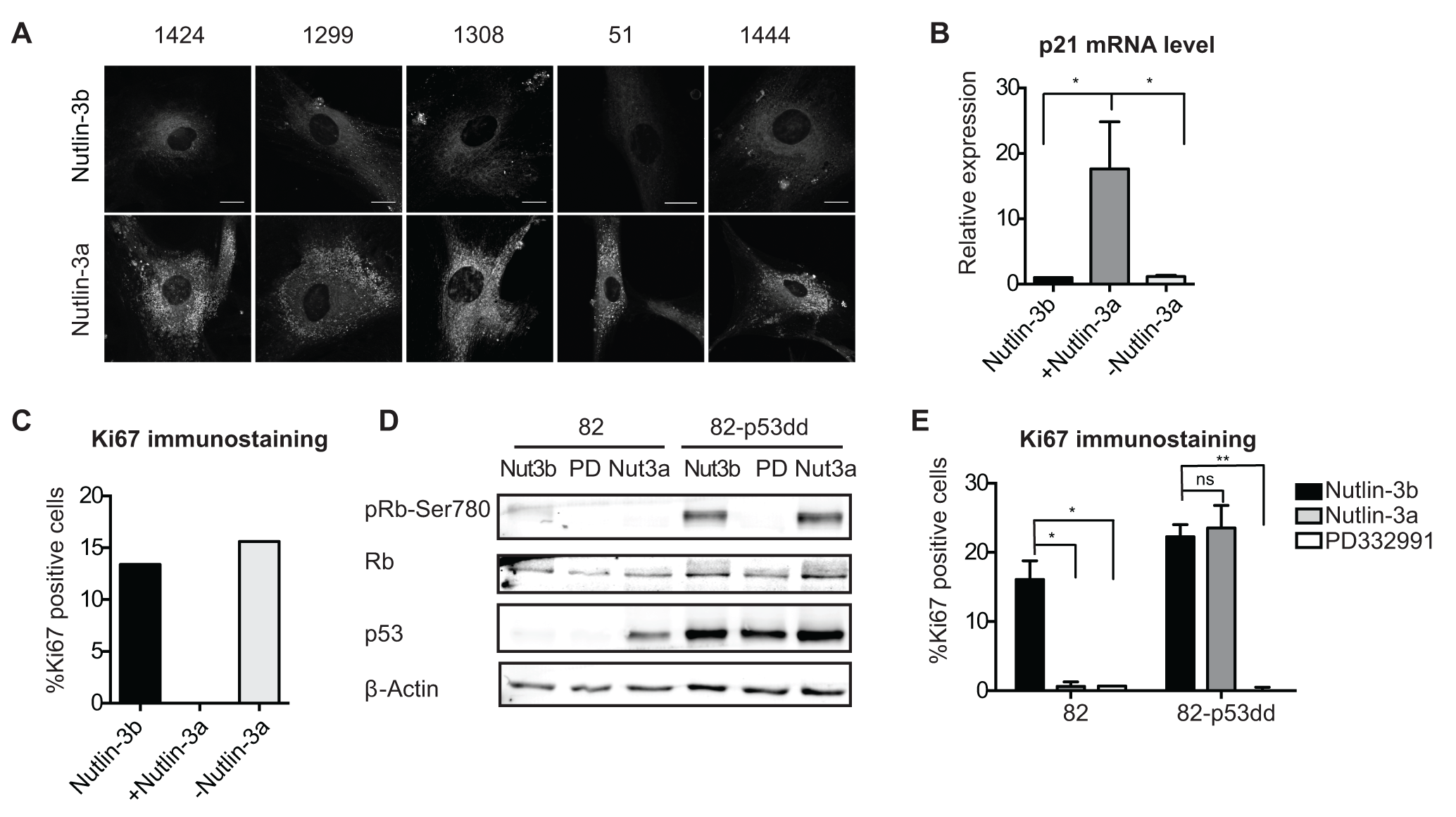

Supplement: S1 Fig — (A) Representative images of caPSCs stained with BODIPY 493/503. (B-C) Cells were treated as described in Fig 2. (B) p21 mRNA level was quantified by RT-qPCR. (C) Bar graph indicates the percentage of nuclei positive for the antigen Ki67. (D-E) caPSCs were treated for 72h with Nutlin-3b, Nutlin-3a or PD332991 (D) Immunoblot of the indicated proteins. β-Actin serves as a loading control. (E) Bar graph indicates the percentage of cells positive for the antigen Ki67. Bar graphs are represented as in Fig 2. Scale bar, 25 μM. (TIF) [file pone.0189051.s001.tif]

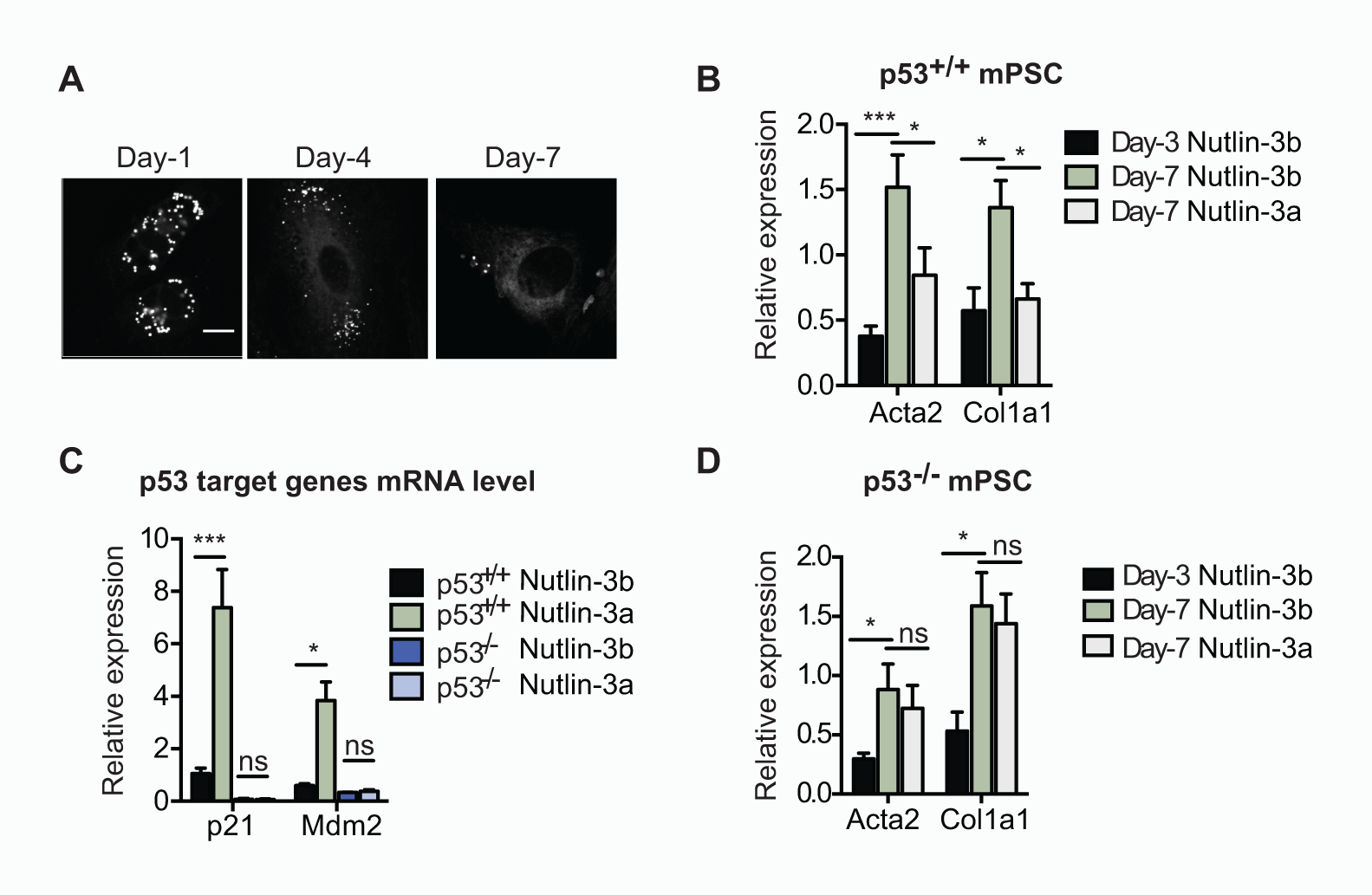

Supplement: S2 Fig — (A) Primary mouse PSCs were isolated from pancreata of wild-type C57B6/J mice and stained with BODIPY 493/503 on days 1, 4 and 7 of culture. Representative images are shown. Scale bar, 10 μM. (B-D) Primary mouse PSCs isolated from pancreata of p53 wild-type (p53+/+) or p53 knock-out (p53-/-) mice were treated with Nutlin-3a or Nutlin-3b and harvested on days 3 and 7 of culture. mRNA levels of the indicated genes were assessed by RT-qPCR and normalized to Rplp0 mRNA. Bars represent mean + SEM of 5 experiments. ***, p<0.001; *, p<0.05 by two-way ANOVA. (TIF) [file pone.0189051.s002.tif]

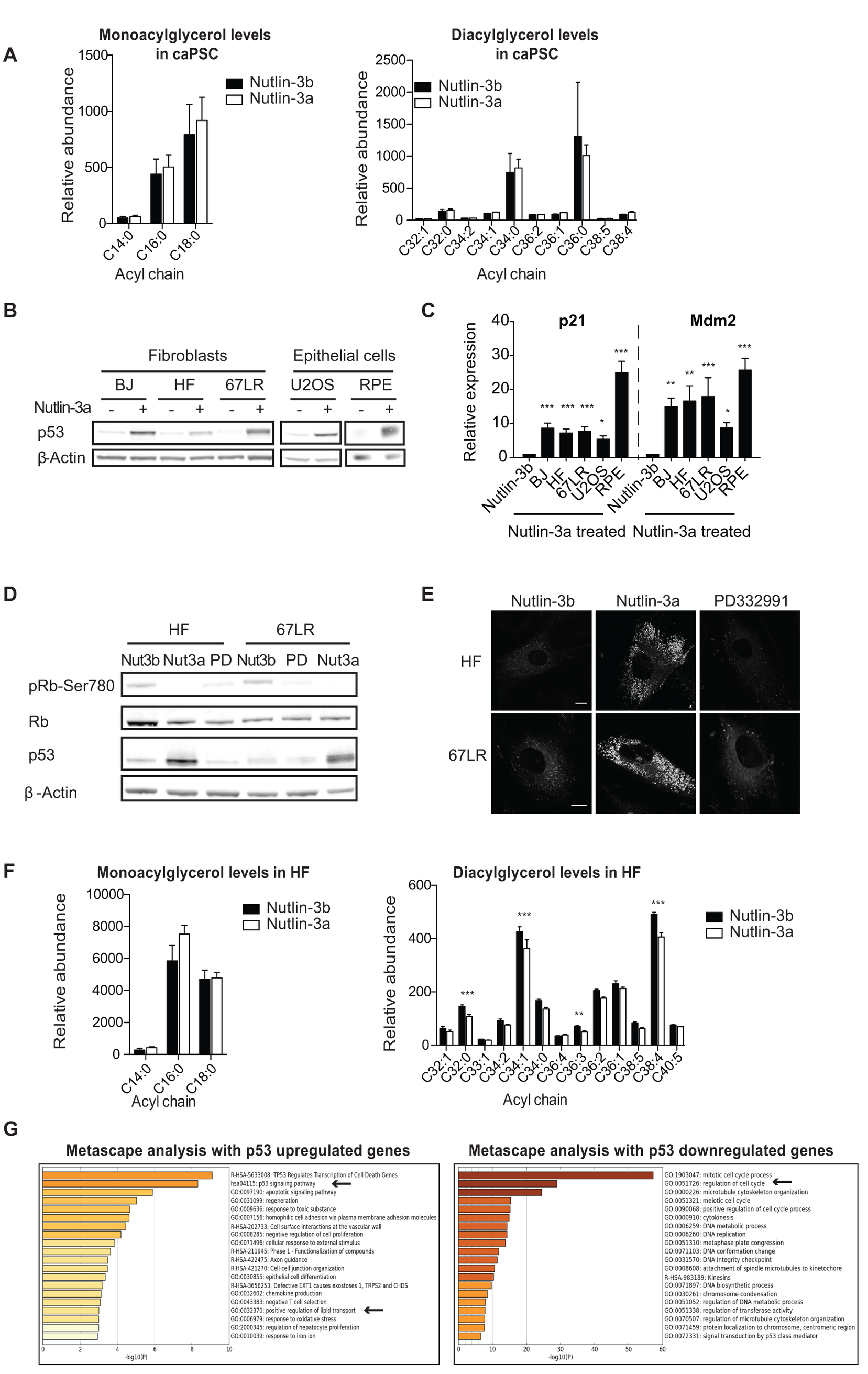

Supplement: S3 Fig — (A) Relative abundance of selected lipids from mass spectrometry-based lipidomic analysis of caPSC-82 treated for 72h with Nutlin-3a or Nutlin-3b, represented as in Fig 4. (B-C) Cells were treated for 48h with Nutlin-3b (-) or Nutlin-3a (+). (B) Immunoblot for p53, β-Actin serves as a loading control. (C) p21 and Mdm2 mRNA levels were quantified by RT-qPCR. Values were normalized to Rplp0 mRNA levels and are represented as fold change relative to Nutlin-3b treated cells. Bars indicate mean +SD of at least 2 experiments. ***, p<0.001; **, p<0.01; *, p<0.05 by one-way ANOVA. (D-E) The skin fibroblast lines HF and 67LR were treated for 72h with Nutlin-3b (Nut3b), Nutlin-3a (Nut3a) or PD332991 (PD). (D) Immunoblot for the indicated proteins. β-Actin serves as a loading control; (E) Representative images of cells stained with BODIPY 493/503. Scale bar, 10 μM. (F) Relative abundance of selected lipids from mass spectrometry-based lipidomic analysis of the skin fibroblast line HF treated for 72h with Nutlin-3a or Nutlin-3b, represented as in Fig 4. (G) Genes regulated in both caPSCs and skin fibroblasts (Nutlin-3a vs Nutlin-3b, adjusted p<0.05, fold-change>2 or < 0.5) were analyzed with Metascape. The 20 most significant canonical pathways are shown for p53 upregulated genes (left) and downregulated genes (right). (TIF) [file pone.0189051.s003.tif]

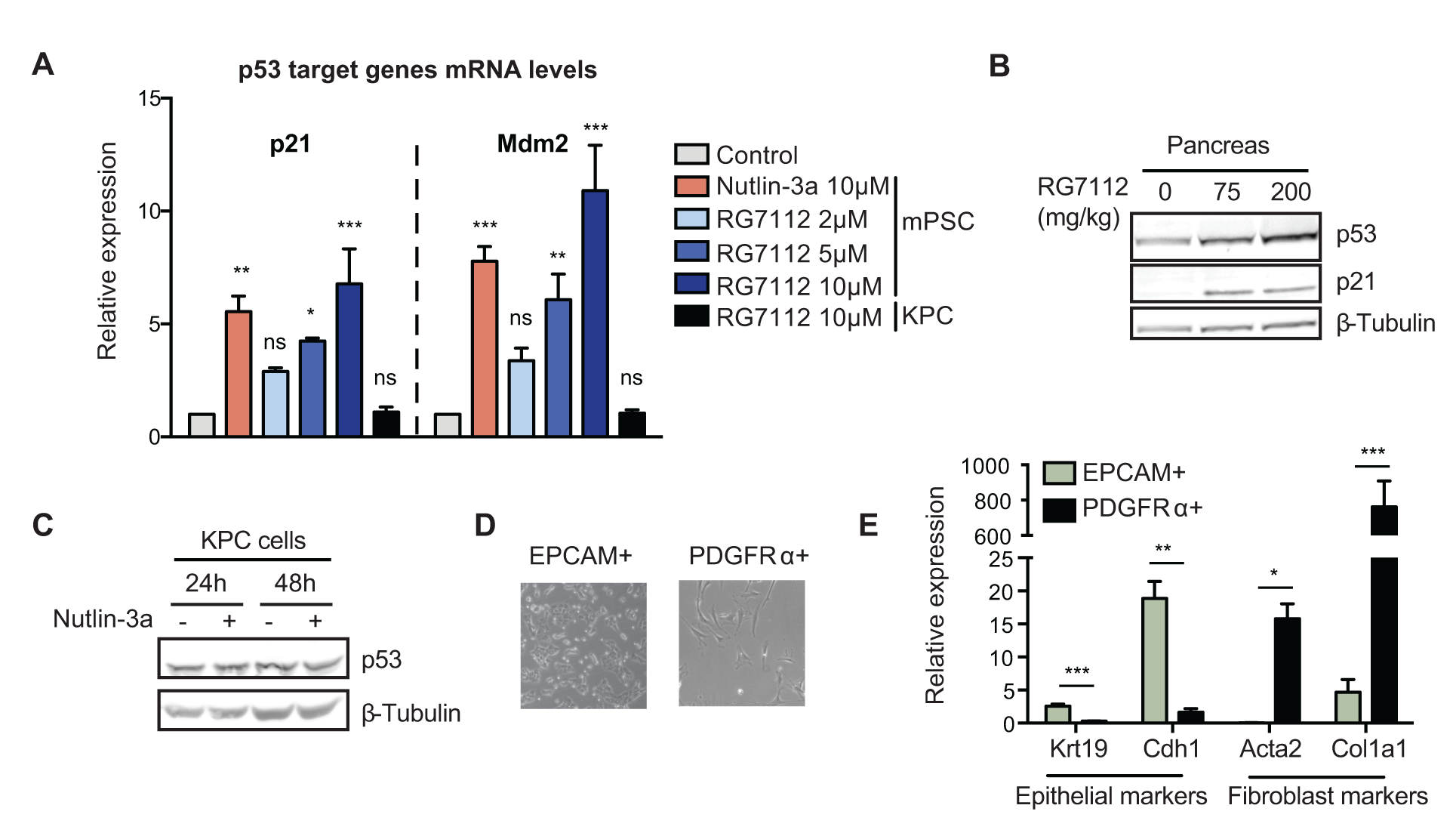

Supplement: S4 Fig — (A) mPSC and KPC cells were treated with Nutlin-3a, RG7112 or control compounds (inactive enantiomers) for 48h. Mdm2 and p21 mRNA levels were assessed by RT-qPCR. Values were normalized to Rplp0 mRNA levels and are represented as fold change relative to the control. Bars indicate mean +SD of 2 experiments. **, p<0.01; *, p<0.05 by one-way ANOVA. (B) Wild-type C57B6/J mice were treated with RG7112 (75 or 200 mg/kg) or vehicle and pancreata were harvested 24h later. p53 and p21 protein levels were analyzed by Western-blot. β-Tubulin serves as a loading control. (C) Immunoblot for p53 from KPC cells treated for 24h and 48h with Nutlin-3a (+) or Nutlin-3b (-). β-Tubulin serves as a loading control. (D-E) Tumors were harvested from transplanted mice and dissociated. EPCAM+ and PDGFRα+ cells were isolated as described in Fig 5. (D) Representative pictures of day 3 of culture. (E) mRNA levels of the indicated genes were assessed by RT-qPCR and normalized to Rplp0 mRNA levels. Mean +SEM for at least 3 mice were plotted. ***, p<0.001; **, p<0.01; *, p<0.05 by Student’s test. (TIF) [file pone.0189051.s004.tif]

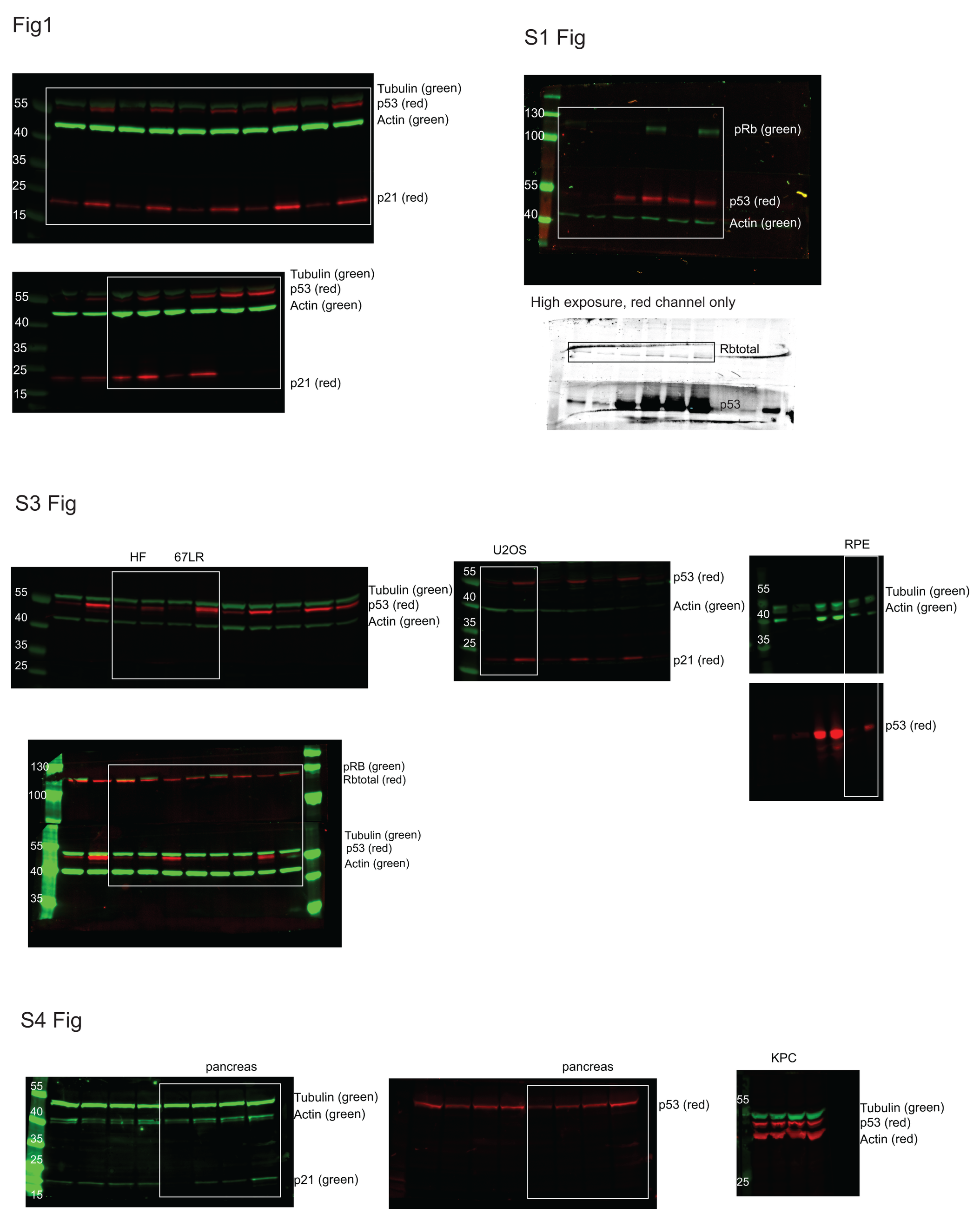

Supplement: S5 Fig — (TIF) [file pone.0189051.s005.tif]
