# Supplementary material for: Reprogramming pancreatic stellate cells via p53 activation: A putative target for pancreatic cancer therapy
Source: PLoS One. 2017 Dec 6;12(12):e0189051. doi: 10.1371/journal.pone.0189051 (PMC5718507; doi:10.1371/journal.pone.0189051)
Supplement: S1 Table — (PDF) [file pone.0189051.s006.pdf]

Table S1. List of primers used for real-time qPCR

		Forward	Reverse
	Rplp0	GTGCTGATGGGCAAGAAC	AGGTCCTCCTTGGTGAAC
	ACTA2	CGATGCTCCCAGGGCTGTTT	TTCGTCACCCACGTAGCTGTCTTT
	MDM2	CTGTGTGTAATAAGGGAGATATGTTGT	GAATGTTCACTTACACCAGCATCAA
**Human**	P21	GGCAGACCAGCATGACAGATT	GCGGATTAGGGCTTCCTCTT
	Acta2	ACTGGGACGACATGGAAAAG	
	Col1a1	ACGCATGGCCAAGAAGAC	GGTTTCCACGTCTCACCATT
	Mdm2	CTGCTCTCACTCAGCGATGT	TCTGTGAAGGAGCACAGGAA
**Mouse**	p21	GGCCCGGAACATCTCAGG	AAATCTGTCAGGCTGGTCTGC
